# Lectin-Mimicking Aptamer as a Generic Glycan Receptor for Sensitive Detection of Glycoproteins Associated with Cancer

**DOI:** 10.1021/acs.analchem.3c05891

**Published:** 2024-02-08

**Authors:** Inés Díaz-Martínez, Rebeca Miranda-Castro, Noemí de-los-Santos-Álvarez, María Jesús Lobo-Castañón

**Affiliations:** † Departamento de Química Física y Analítica. Universidad de Oviedo, Av. Julián Clavería 8, 33006 Oviedo, Spain; ‡ Instituto de Investigación Sanitaria del Principado de Asturias, Av. de Roma, 33011 Oviedo, Spain

## Abstract

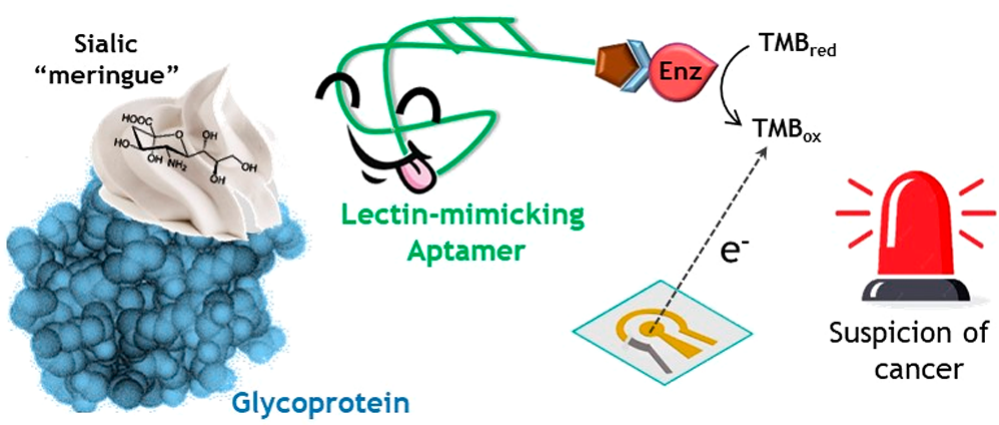

The shortage of specific glycan recognition reagents has proven a significant hurdle in the development of assays to detect altered glycoforms associated with cancer. Here, a carbohydrate-binding aptamer originally selected against the glycan moiety of prostate-specific antigen (PSA) is used as a lectin-mimicking reagent. As a first proof-of-principle, this aptamer has been applied to develop a sandwich-type electrochemical biosensor for the detection of the serum amyloid P (SAP) component, a glycosylated protein whose increased sialylation has been associated with pancreatic cancer. The assay combines a specific antibody for this potential tumor biomarker and the aptamer as capture and detection receptors, respectively. Two oriented antibody immobilization approaches, protein A-based and boronic ester-based attachment to self-assembled monolayers built onto gold surfaces, were comparatively evaluated, the latter being able to circumvent the unwanted interaction between the aptamer and the glycans on the electrode-attached antibody. The resulting biosensing platform allows the detection of the SAP glycoprotein at levels of nanograms per milliliter with a reproducibility value lower than 20%, both in aqueous buffer and in serum. This work represents a proof-of-concept of a promiscuous ligand of proteins with high levels of sialylated glycans typically produced by cancer cells.

To become fully functional, human proteins resulting from mRNA translation undergo a series of modifications known as post-translational modifications or PTMs. These relevant nongenetically encoded modifications are mediated by enzymes and encompass, among others, phosphorylation, acetylation, and glycosylation. Protein glycosylation involves the addition of simple or branched saccharides (i.e., glycans) to proteins, giving rise to two main categories: *N*-glycans (attached to asparagine residues by a covalent bond) and *O*-glycans (covalently linked to serine or threonine residues), the first being the most common glycosidic linkage.^1^

Alterations in the protein glycosylation pattern, including sialylation, fucosylation, *N*- and *O*-linked glycan branchings, and *O*-glycan truncation, have been related to the onset and progression of different types of cancer.^2,3^ Strikingly, even if many serum cancer biomarkers approved by the FDA agency are glycoproteins,^4^ only their total protein levels are clinically monitored through ELISA tests at the expense of compromised clinical specificity and sensitivity (significant false positives and negatives).^5,6^ The capability to detect cancer by targeting abnormal protein glycosylation has the potential to boost the efficiency of cancer management. Therefore, there is a clear need for ligands capable of binding a specific glycoform of a particular protein. Receptors with binary recognition of a glycoprotein, those simultaneously binding the peptide region and the altered glycan moiety, are the ideal goal but are particularly difficult to obtain.^7−9^

An alternative option is to combine two affinity reagents in a sandwich format assay, one to capture the protein of interest, typically an antibody, and another to detect specific glycan epitopes, thus discriminating disease-related from healthy protein glycoforms. Different glycan-binding proteins, also referred to as glycan “readers”,^10^ have been studied so far.^11^ Lectins show high selectivity toward defined glycosidic bonds, and they are widely used as receptors for glycan identification. However, their overall affinity is modest (μM–mM *K*_d_ values), only circumvented by multivalent complexes formation.^12^ Antiglycan antibodies can exhibit high affinity, but the low immunogenicity of carbohydrates hampers their generation.^13^ As artificial nucleic acid-based receptors, aptamers are particularly attractive by offering reproducible and affordable synthesis, thermal and chemical stability, and amenability to chemical modification, while allowing the targeting of their selection toward the domain of interest in a simpler and more efficient way than in the case of antibodies production.^14^ As a result, several glycoreactive aptamers have been reported.^13,15,16^

We have evolved a DNA aptamer against the glycan moiety of prostate-specific antigen (PSA), the so-called PSA-1, capable of distinguishing the natural glycosylated protein and its recombinant unglycosylated counterpart.^17^ Through selective PSA deglycosylation assays, it was concluded that the PSA-1 aptamer recognizes external sugars, mainly sialic acids and galactoses, but not the peptide region.^7^ Consequently, this aptamer could be used as a generic receptor for the detection of glycans on other proteins, thus emulating the natural receptors, lectins, and antiglycan antibodies, but with the aforementioned strengths of these synthetic receptors.

Thus, motivated, we set out to ascertain whether the PSA-1 aptamer could be used in the development of a biosensor for the detection of glycan-based cancer biomarkers other than PSA. We focus our efforts on pancreatic ductal adenocarcinoma, PDAC, an aggressive tumor with dismal prognosis,^18,19^ which is predicted to become the second leading cause of cancer-related death by 2040.^20^ In the search for accurate biomarkers for the early, minimally invasive diagnosis of PDAC, increased fucosylation and sialylation in certain glycoproteins such as the serum amyloid P (SAP) component has been reported in serum samples of pancreatic cancer patients when compared to those from noncancerous controls and individuals with acute pancreatitis.^21^ The glycosylated SAP protein was recognized by *Sambucus nigra* (SNA) lectin, which preferentially binds to sialic acids attached to terminal galactose in an α-2,6 linkage and, to a lesser extent, an α-2,3 linkage. Bearing in mind the selectivity of this lectin toward the cancerous glycoform of the SAP protein and the experimental evidence pointing to the recognition of sialic acid-terminated glycans by the PSA-1 aptamer, we envisage the replacement of the SNA lectin by the PSA-1 aptamer for the development of an electrochemical biosensor for SAP detection in human serum samples, involving an anti-SAP antibody immobilized onto the transducer surface along with the glycan-binder PSA-1 aptamer in a sandwich configuration.

A crucial point that directly impacts biosensor performance is the immobilization of the bioreceptor. To avoid impairing their antigen-binding capacity, the oriented immobilization of antibodies onto the solid support is preferable. In this regard, protein A is very convenient since it recognizes the Fc region of an antibody, while the Fab region containing the paratope remains fully accessible to the antigen.^22^ Protein A is attached to a carboxyl-functionalized gold electrode by the covalent coupling of its primary amino groups, resulting in an organized biosensing architecture.

The protocol used to modify the gold electrodes with protein A for subsequent anchoring of the anti-SAP antibody is detailed in the Supporting Information. Briefly, a mixed self-assembled monolayer of thiols 11-mercaptoundecanoic acid (MUA) and 6-mercapto-1-hexanol (MH) is constructed on the surface of the previously conditioned gold working electrode. Next, the carboxylic acid groups of MUA are activated with carbodiimide and *N*-hydroxysuccinimide for the subsequent immobilization of protein A through its amino groups. After blocking the carboxylic groups that have not reacted with ethanolamine, the anti-SAP antibody is immobilized (Figure 1A).

**Figure 1:**
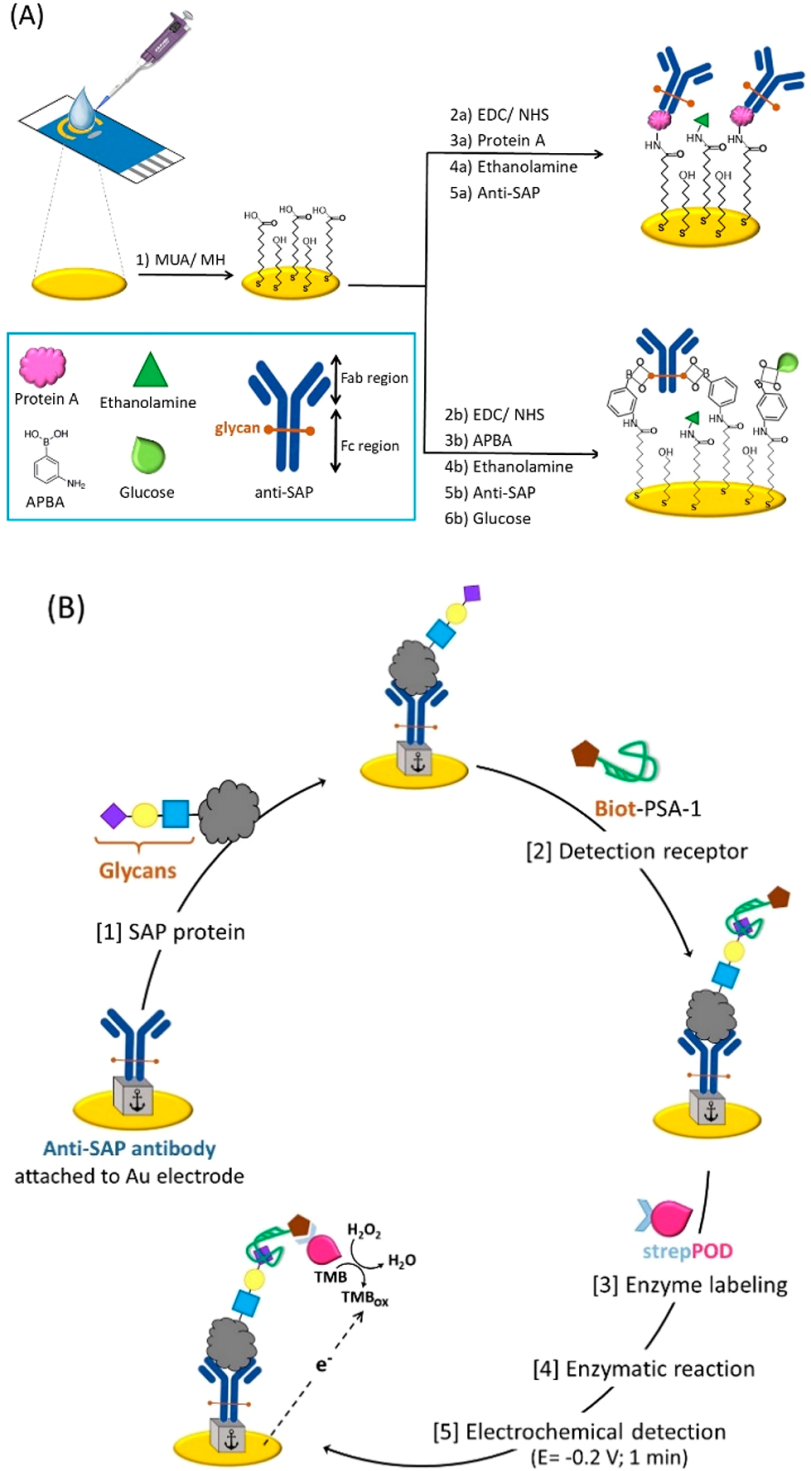
(A) Strategies for oriented antibody immobilization: protein A-based and boronic ester-based attachments to carboxyl-terminated self-assembled monolayers onto gold electrodes. (B) Schematic illustration of the electrochemical sandwich assay on screen-printed gold electrodes.

To evaluate the feasibility of this strategy, a mixed sandwich assay was performed as described in the Supporting Information and schematized in Figure 1B. First, the sensing phase was challenged with variable amounts of SAP protein from a commercial cell lysate and then incubated with an excess (1 μM) of the PSA-1 aptamer that is labeled with biotin. Next, a labeling step with the streptavidin-peroxidase enzyme conjugate (strep-POD) was carried out. Finally, to determine the enzymatic activity immobilized on the sensing phase, which is directly related to the amount of SAP protein, the enzyme substrates (H_2_O_2_ + TMB_Red_) were incorporated onto the biosensing surface to conduct the enzymatic reaction during a controlled time of 30 s. The amount of enzymatically generated TMB_Ox_ was quantified by chronoamperometry by applying a potential of −0.2 V to the working electrode for 1 min. The average of the current intensity recorded during the last 10 s in absolute value is used as the analytical signal.

As can be seen from Figure 2A, the measured signal increases with the protein concentration, indicating that the PSA-1 aptamer is capable of binding to the SAP protein. But despite the signal differences in the absence and presence of serum amyloid protein P, the blank signal remains relatively high (average value of almost 1 μA).

**Figure 2:**
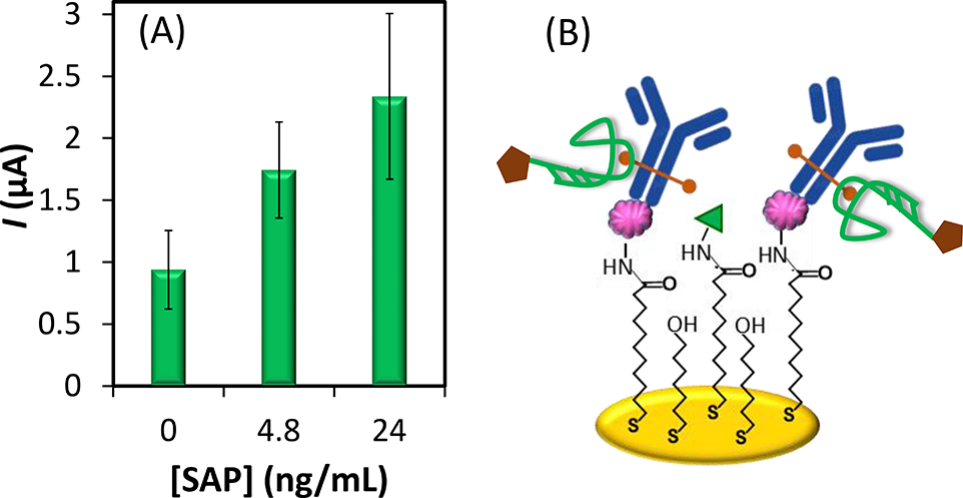
(A) Electrochemical response of the mixed sandwich biosensor resulting from the protein A-based immobilization of the capture anti-SAP antibody. (B) Schematic illustration of the possible source of high background signal.

Since the capture antibody belongs to the G class of immunoglobulins, it is *N*-glycosylated at asparagine 297 of the CH2 domains of the Fc region.^23^ Thus, it is possible the PSA-1 aptamer binds to this glycosylated region of the immobilized anti-SAP antibody, as illustrated in Figure 2B. This hypothesis is in line with the need to chemically block *N*-glycans on antibody microarrays to prevent their binding to the lectins used to identify protein glycoforms.^24^ In order to reduce the recorded current intensity that is unrelated to the SAP protein amount, we explored an alternative antibody immobilization approach. Pursuing the original idea of an oriented immobilization of the protein receptor and trying to circumvent the likely nonspecific interaction of the PSA-1 aptamer with the carbohydrates of the capture antibody, the possibility of anchoring the anti-SAP antibody through its sugars was then evaluated. To address this challenge, the characteristic chemistry of the vicinal diols located within the *N*-glycans attached to the antibody was exploited, more specifically, the reaction of cis-diol groups with surface-anchored boronic acids to form boronic esters (Figure 1A). This strategy has several key benefits:^25^ (a) The antibody binding sites remain free and oriented away from the sensing surface. (b) It does not require prior modification of the antibody. (c) It is cheaper when compared to the binding of the Fc region to protein A-modified surfaces. The boronic ester-based immobilization of the antibody consists of the formation of a mixed self-assembled monolayer of the MUA and MH thiols onto the gold working surface for subsequent attachment of the amino boronic acid derivative 3-aminophenylboronic acid (3-APBA) to the carboxyl groups of MUA through the carbodiimide reaction. After blocking with ethanolamine, the activated carboxylic groups of MUA that did not bind 3-APBA, the anti-SAP antibody was immobilized. Finally, to avoid the undesired binding of the sugars from SAP protein to free 3-APBA, a glucose-blocking step was carried out (Figure 1A). With this approach, well-defined points of anti-SAP attachment as well as good accessibility of the antigen-binding sites on the antibody are attained while avoiding unwanted binding (blank signals of 0.19 ± 0.05 μA).

When the response of the electrochemical mixed aptamer–antibody sandwich assay was evaluated against different amounts of SAP protein (Figure 3A), a linear correlation could be established (R^2^ = 0.992) for a dynamic protein concentration range from 10 to 100 ng/mL (*I*_net_, μA = 0.027 (±0.002) × [SAP, ng/mL] + 0.05 (±0.07); n = 5), with a reproducibility of 18% estimated as the average relative standard deviation. A decrease in the analytical signal was recorded when the analyte concentration was expanded to 250 ng/mL, which points to the so-called hook effect (Figure 3B). The limit of detection, LOD, calculated as three times the standard deviation of the y-intercept of the regression equation divided by its slope, turned out to be 8.2 ng/mL (0.32 nM assuming a SAP molecular weight of 25.4 kDa).

**Figure 3:**
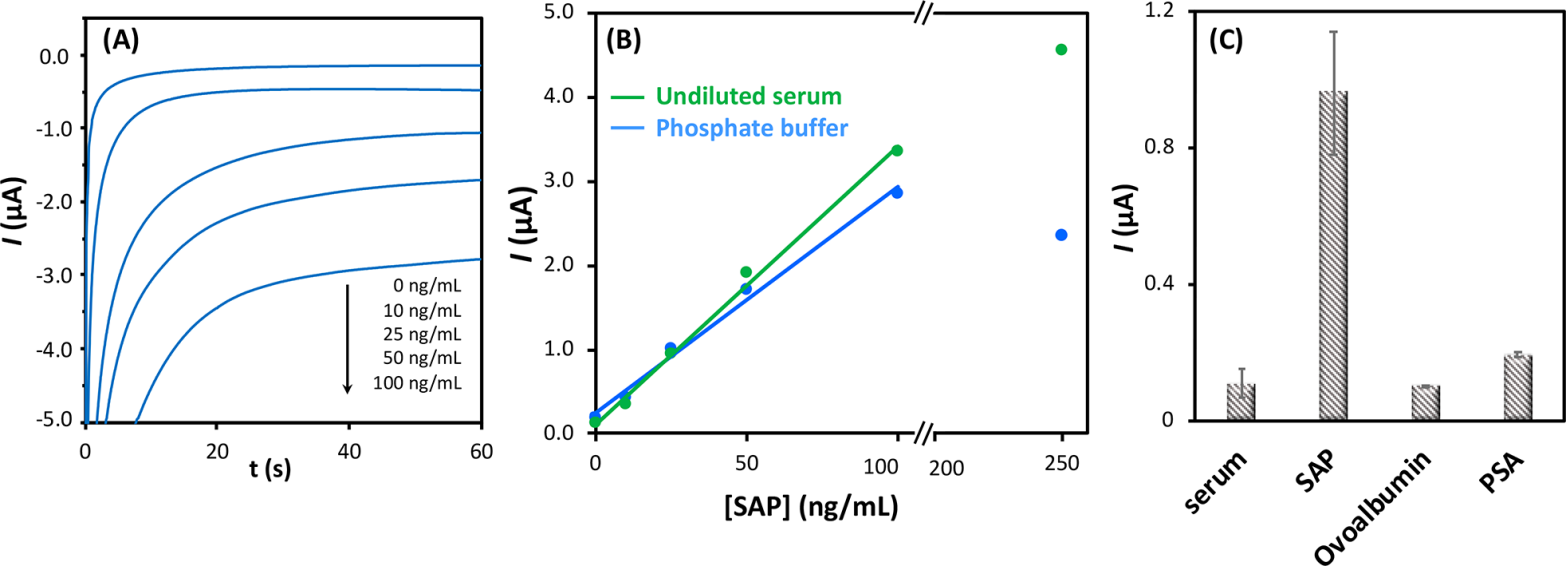
Chronoamperometric sensing platform involving boronic ester-based immobilization of anti-SAP antibody as the capture receptor and PSA-1 aptamer as the detection receptor. (A) Recorded chronoamperograms for variable SAP protein concentrations in phosphate buffer. (B) Calibration plots of SAP protein in phosphate buffer (blue) and in human female serum (green). (C) Sensor response to the glycoproteins: SAP (25 ng/mL), ovalbumin (25 ng/mL), and PSA (10 ng/mL) in serum. Error bars correspond to the relative standard deviations of three independent experiments.

Subsequently, the cross-reactivity was evaluated against two glycoproteins: PSA and ovalbumin (Figure 3C). PSA was selected as a potential interfering species because it was employed as a target in the SELEX process leading to the PSA-1 aptamer, whereas ovalbumin harbors an *N*-linked glycosylation site and has a higher molecular weight.

For that, a pool of human female serum samples whose levels of PSA protein were negligible (<0.01 ng/mL with the ELISA assay) were spiked with both proteins. The current intensity measured for female serum supplemented with PSA at 10 ng/mL was statistically different from the background signal, although it corresponds to a SAP concentration lower than the calculated detection limit of the platform, thus confirming its ability to distinguish between SAP and PSA proteins. In the case of ovalbumin, the signal recorded for 25 ng/mL was indistinguishable, considering the experimental error, from that obtained for the background (human serum) and negligible compared to that for the same concentration of the SAP protein (Figure 3C).

The assay selectivity was further investigated by challenging the sensing platform to measure the SAP protein concentration spiked in human serum. Remarkably, the sensor results in undiluted serum fortified with SAP were slightly better than those obtained in buffer (Figure 3B and Table S1), albeit without an appreciable hook effect across the stated range and with a slight improvement in the LOD (6.4 ng/mL), probably due to a lower background signal.

The results described above evidence the proper performance of the hybrid antibody–aptamer sandwich electrobioassay for SAP glycoprotein detection in unprocessed human serum (i.e., there is no depletion of abundant proteins, just a minimum dilution for SAP fortification) without the interference of PSA, the target glycoprotein used in the *in vitro* selection process of the PSA-1 aptamer.

Despite not yet being well established at what levels it would be necessary to detect the SAP protein in human serum,^21^ the methodology described involving the PSA-1 aptamer has proven to be selective, and the oligonucleotide nature of this detection receptor would enable, if necessary, the implementation of amplification strategies based on nucleic acids.^26^

The method developed herein has the potential to be generalized and applied to the detection of other human sialylated glycoproteins of clinical relevance with the aid of both an unlabeled specific antibody raised against the core protein of the target molecule and the biotinylated PSA-1 aptamer. Besides the evident case of PSA, serving as biomarker for prostate cancer screening, according to our previous results,^17^ we could anticipate a good performance when implemented for neutrophil gelatinase-associated lipocalin (NGAL, also known as LCN-2), a single *N*-glycosylated protein similar in size to PSA (28.7 and 22.6 kDa for PSA and NGAL, respectively). Computational studies of the aptamer–PSA glycoprotein complex are currently in progress to decipher the glycan epitope targeted by the PSA-1 aptamer and thereby to extend its usefulness as a receptor of cancer-associated glycans.

## Supporting Information Available

Reagents, apparatus, experimental protocols, and table with the analytical performance of the antibody–aptamer biosensor (PDF)
