# Supplementary material for: Lectin-Mimicking Aptamer as a Generic Glycan Receptor for Sensitive Detection of Glycoproteins Associated with Cancer
Source: Anal Chem. 2024 Feb 8;96(7):2759–63. doi: 10.1021/acs.analchem.3c05891 (PMC10882573; doi:10.1021/acs.analchem.3c05891)
Supplement: Supplementary file 1 — ac3c05891_si_001.pdf [file ac3c05891_si_001.pdf]

Electronic Supplementary Information

Lectin-Mimicking Aptamer as a Generic Glycan Receptor for Sensitive Detection of Glycoproteins Associated with Cancer

Inés Díaz-Martínez[a], Rebeca Miranda-Castro[a,b*], Noemí de-los-Santos-Álvarez[a,b], María Jesús Lobo-Castañón[a,b*]

[a]Departamento de Química Física y Analítica. Universidad de Oviedo, Av. Julián Clavería 8 33006 Oviedo, Spain.

[b]Instituto de Investigación Sanitaria del Principado de Asturias, Avenida de Roma 33011 Oviedo, Spain.

* corresponding authors:

RMC: mirandarebeca@uniovi.es

MJLC: mjlc@uniovi.es

Table of Contents

Experimental Section

Reagents……………………………………………………………………….......................................S3

Apparatus …………………………...…………..S3

Experimental protocols………………………………………………………………………………………S4

Reconstitution and quantification of SAP protein……………….………………………………S4

Electrode conditioning……………………………………………………………………..……..S5

Protein A-based immobilization of the capture antibody…………………………………S5

Boronic ester-based immobilization of the capture antibody……………………………S6

Mixed sandwich assay for SAP protein………………………………………………………….S6

Supplementary Tables

Table S1. Analytical performance of the antibody-aptamer biosensors……………………..…..S7

Experimental

Reagents

Human SAP glycoprotein in the form of a lyophilized cell lysate was purchased from Lifespan Biosciences (WA, USA). The ELISA kit for the quantification of SAP protein (SEB539Hu-24T) from Cloud-Clone Corporation (USA) was provided by bioNova Científica (Spain). The anti-SAP capture antibody (ab27313) used in the electrochemical assay was supplied by Abcam (UK). The PSA-1 aptamer whose 5' end is labeled with biotin (Biotin-GGA CGG TTG CGC TAT ATT TAA CCA AAA GTC TGG ATT AAC A) was supplied by Metabion (Germany) in lyophilized format. 6-mercapto-1-hexanol (MH), 11-mercaptoundecanoic acid (MUA), 1-ethyl-3-(3-dimethylaminipropyl)carbodiimide (EDC), N-hydroxysuccinimide (NHS), ethanolamine, 3,3´,5,5´-tetramethylbenzidine (TMB_{Red}) in a format that includes H_2O_2, 3-aminophenylboronic acid (3-APBA), and D(+)-glucose were purchased from Merck/Sigma-Aldrich (Spain). The peroxidase enzyme conjugated to streptavidin (strep-POD) and protein A were purchased from Thermo Fisher Scientific (Spain). Glycoproteins used in selectivity studies, ovalbumin and prostate-specific antigen (PSA) from human seminal fluid (P117-7), were obtained from Merck/Sigma-Aldrich (Spain) and from BBI Solutions (UK), respectively. The serum sample used was a pool of leftover sera from women subjected to routine analysis at Cabueñes hospital. The hospital laboratory established through an ELISA that the PSA levels in this pool were below the LOD. SAP levels as measured with the commercial ELISA were also below LOD.

Reagents needed to prepare the buffer solutions used: 1× PBS (0.1 M phosphate, 0.154 M NaCl pH 7.4), sodium acetate or NaAc (10 mM pH 5.5), and HEPES (20 mM pH 8.5) were purchased from Merck/Sigma-Aldrich, while ethanol (96%) and concentrate sulfuric acid were supplied by J.T. Baker. Ultrapure water with a resistivity of 18.2 MΩ was obtained from a Milli-Q water purification system and used throughout the experiments.

Apparatus

Screen-printed electrochemical cells with 12.6 mm^2 gold working electrodes (SPC, DRP-220BT) were supplied by Metrohm-DropSens (Spain). A DRP-DSC connector (Metrohm-DropSens, Spain) is also required as interface between the SPC and the potentiostat.

Electrochemical measurements were carried out using a μ-AutoLab type II Eco Chemie B.V. (NOVA 2.1. software) potentiostat with a three-electrode system featuring the core screen-printed Au electrode as the working electrode, Ag|AgCl|KCl (3M) (or Ag-SPC) as the (pseudo)reference electrode and a platinum wire (or Au-SPC) as the counter electrode.

Protocols

Reconstitution and quantification of SAP protein

The cell lysate containing the overexpressed SAP protein was reconstituted following the manufacturer's guidelines. Briefly, the vial was centrifuged at 5000 rpm for 15 min to recover the lyophilisate. Subsequently, 200 µL of deionized water was added, gently shaken, and boiled for five minutes. Considering that the amount supplied was 300 µg, the concentration of the reconstituted cell lysate is 1500 µg/mL. Once reconstituted, the lyophilisate was aliquoted and stored at -80°C for long-term use and at 4°C for immediate use.

To determine SAP protein concentration in the reconstituted lyophilisate, an ELISA kit designed to quantify this protein in serum, plasma, tissues and other biological fluids was employed. It is a sandwich format immunoassay in which two antibodies participate: a capture antibody adsorbed on the surface of the support where the assay is conducted and a biotinylated detection antibody. The enzyme peroxidase (POD) serves as the enzymatic tag incorporated through the biotin-avidin affinity reaction. The enzymatic activity specifically immobilized on the wells is spectrophotometrically measured at 450 nm, after 10 minutes of POD-catalyzed oxidation of TMB_{Red} with hydrogen peroxide, followed by the addition of sulfuric acid to stop the enzymatic reaction.

The experimental data recorded for serial dilutions of the SAP standard fitted well to the following equation: $y = \frac{y_{max} \cdot x}{C_{50}+x}$; where *y* is the absorbance at 450 nm, *x* the protein concentration in nM, y_{max} the maximum absorbance value corresponding to the saturation of the capture antibody binding sites, and C_{50} the protein concentration for which the recorded absorbance is half the maximum value. More specifically, the values of 6.9 ± 0.8 a.u. and 4.5 ± 1.0 nM were obtained for y_{max} and C_{50}, respectively, with a correlation coefficient of 0.995.

The amount of SAP protein in the lyophilisate solution was determined by interpolation in the previous equation. For its calculation it was kept in mind that the ELISA kit standard is an unglycosylated fragment of 159 amino acids (Ala65-Val223), while the complete glycoprotein present in the commercial lyophilisate consists of 223 amino acids (Met1-Val223) with a molecular weight of 25,387 Da (https://www.glygen.org/protein/P02743-1).

Electrode conditioning

The screen-printed electrochemical cells (SPCs) were first cleansed with ethanol and deionized water, and then dried under a nitrogen stream. Subsequently, the working electrodes were electrochemically polished in 0.5 M H_2SO_4 by running 10 scans in the potential window from 0 V to 1.3 V (*versus* Ag-SPC) at 100 mV/s, thus obtaining a stable cyclic voltammogram. Finally, the screen-printed electrochemical cells were washed with water and dried with nitrogen gas just before the formation of the sensing surface.

Protein A-based immobilization of the capture antibody

Following the conditioning step, the screen-printed gold working electrodes were modified with 10 µL of a 3:1 solution of 6-mercapto-1-hexanol (MH): 11-mercaptoundecanoic acid (MUA) to construct a mixed self-assembled monolayer (SAM). For this, 1 mM solutions of each thiol were prepared in a 10 mM sodium acetate (NaAc) solution (pH 5.5), obtained from 100 mM solutions in ethanol (96%). The mixture was incubated on the gold working electrodes overnight at 4 °C in an atmosphere with controlled humidity to ensure the correct formation and organization of the monolayer and to avoid drop evaporation. (Figure 1A, stage 1). The carboxylic acid functionalized SAM was subsequently activated by using the carbodiimide attachment chemistry. With such a purpose, a mixture of 100 mM 1-ethyl-3-(3-dimethylaminipropyl)carbodiimide hydrochloride (EDC) and 25 mM N-hydroxysuccinimide (NHS) was freshly prepared in water, and 10 µL of this were placed on the working electrode and incubated for 30 min at room temperature (RT), generating a succinimidyl ester-terminated SAM. (Figure 1A, stage 2a). After that, the working surface was covered with 10 µL of 250 µg/mL protein A prepared in NaAc (pH 5.5) for 30 minutes at RT, thus achieving its covalent bond through an amide coupling. (Figure 1A, stage 3a). The activated carboxyl groups that did not bind to protein A must be blocked to prevent non-specific binding. The blocking step was carried out with 1 M ethanolamine in 1x PBS, allowing it to react for 15 min at RT (Figure 1A, stage 4a). Finally, the protein A-functionalized working electrode surface was incubated for 30 min at RT with 10 µL of a solution of the anti-SAP capture antibody in 1× PBS. (Figure 1A, stage 5a)

Boronic ester-based immobilization of the capture antibody

Similar to the protein A-based approach, the clean gold surface was first modified with a mixed self-assembled monolayer (SAM) of the alkanethiols MUA and MH (0.75 mM MH and 0.25 mM MUA in 10 mM NaAc pH 5.5 at 4°C overnight). (Figure 1A, stage 1). The terminal-carboxylic groups of the SAM were then activated using EDC/NHS chemistry (100 mM EDC and 25 mM NHS in water for 30 min). (Figure 1A, stage 2b).

Afterwards, an amide covalent bond was formed by covering the NHS ester-coated working surface with a solution of the amino boronic acid derivative 3-aminophenylboronic acid, 3-APBA, (50 mM 3-APBA in 1× PBS pH 7.4) and incubating for 24 h (Figure 1A, stage 3b). Remaining active esters were then deactivated with a solution of ethanolamine (1 M in 1× PBS pH 7.4, for 15 min), thus preventing the amine groups of the antibody from reacting with the ester groups on the surface and causing a random orientation (Figure 1A, stage 4b). Subsequently, the boronic acid-functionalized surface was incubated with the capture antibody solution (75 µg/mL in 20 mM HEPES pH 8.5) for 1 h (Figure 1A, stage 5b). After anti-SAP antibody site-directed immobilization, the unoccupied binding sites were blocked with D-glucose (30 µg/mL in 1× PBS pH 7.4, for 15 min) to preclude the attachment of unwanted glycocompounds (Figure 1A, stage 6b).

Unless otherwise indicated, 10 µL of each of the previous solutions were placed onto the gold working electrode surface for incubation at room temperature.

Mixed sandwich assay for SAP protein

The procedure for SAP protein quantification using the anti-SAP antibody bound to SPCs functionalized with protein A or 3-aminophenyl boronic acid was as follows. 10 µL of the cell lysate in 1× PBS, containing the SAP protein in the desired concentration, were deposited on the sensing surface and allowed to react for 30 min at room temperature (RT). After that, 1 µM biotin-PSA-1 aptamer prepared in PBS was incubated for 30 min at RT. The enzymatic labeling step was then performed by adding 10 µL of streptavidin-peroxidase conjugate (strep-POD), diluted at 0.5 U/mL of POD in 1× PBS supplemented with 0.5% casein (w/v) as blocking agent. After a 15-min incubation step at RT, the electrochemical cell was covered with 35 µL of the enzyme substrate solution (H_2O_2 & TMB_{Red}) and the enzymatic reaction proceeded at RT for 30 s after which the current intensity was measured by chronoamperometry at -0.2 V for 1 min. After each step, the electrode surface was conditioned with the buffer used in the next step and dried with N_2. Figure 1B illustrates the steps involved in this protocol.

Table S1. Analytical performance of the antibody-aptamer biosensor in phosphate buffer and in undiluted female human serum.

	Phosphate buffer	Undiluted serum
**Sensitivity (µA mL ng^{-1})**	0.027 ± 0.002	0.033 ± 0.001
**Linear response range**	10-100 ng/mL	10-100 ng/mL
**Blank signal (nA)**	194	122
**LOD* (ng mL^{-1})**	8.2	6.4
**RSD (%)**	18.0	18.6

* Calculated as three times the standard deviation of the y-intercept of the regression equation divided by its slope.

Comparing the absolute difference between both calibration slopes, in phosphate buffer and in serum, (Δ_{slope} = 0.006) with its uncertainty ($u_{\Delta slope}$ = 0.002), because $u_{\Delta slope} < \Delta_{slope}$, we conclude that there is significant difference between the calibration slopes.
